# Taxonomic etymology – in search of inspiration

**DOI:** 10.3897/zookeys.513.9873

**Published:** 2015-07-16

**Authors:** Piotr Jóźwiak, Tomasz Rewicz, Krzysztof Pabis

**Affiliations:** 1 Department of Invertebrate Zoology & Hydrobiology, University of Łódź, Łódź 90–237, Poland

**Keywords:** ICZN, taxonomy, mythology, pop-culture, word-play, politics

## Abstract

We present a review of the etymology of zoological taxonomic names with emphasis on the most unusual examples. The names were divided into several categories, starting from the most common – given after morphological features – through inspiration from mythology, legends, and classic literature but also from fictional and nonfictional pop-culture characters (e.g., music, movies or cartoons), science, and politics. A separate category includes zoological names created using word-play and figures of speech such as tautonyms, acronyms, anagrams, and palindromes. Our intention was to give an overview of possibilities of how and where taxonomists can find the inspirations that will be consistent with the ICZN rules and generate more detail afterthought about the naming process itself, the meaningful character of naming, as well as the recognition and understanding of names.

## Introduction

The irresistible desire to classify things has always been an important element of culture and science. In the pre-modern era this resulted in, for example, the creation of a list of angels in Dante’s *Divine Comedy* (14th century), or a catalogue of various curiosities in Caspar Schott’s *Physica curiosa* (17th century). In *The Infinity of Lists* Eco (2009) provides an exhaustive discussion about various catalogues and registers. In the zoological context, it also led to classifications of life forms that resulted in first taxonomical systems. At the same time, the creation of lists is inextricably linked with the process of ‘naming’. Of the two, Foucault (1994) suggested that “*finding words that will at last name accurately that which has never been named before*” is probably the most difficult task.

According to Foucault (1994) “*natural history does not have to establish a system of names based upon representations that are difficult to analyse, but only to derive it from a language that has already been unfolded in the process of description*”, i.e. names are, in a sense, already accessible or derived through the tradition of zoological nomenclature. The examples described in our paper show the difficulties and problems, but also solutions and reasons for decisions hidden behind the process of choosing names for new taxa. The name may also reveal the circumstances under which a given taxon was named and at the same time shows how a name may lead to completely erroneous assumptions about the characteristic of species.

Zoological nomenclature has evolved over the centuries according to any prevailing official system (poly– or binominal), language used, derivation and inspiration hidden behind the names. The first attempts to unify rules of nomenclature and to make it more regular date back to the end of 19^th^ century with, for example, ‘*Rules for Zoological Nomenclature*’ proposed by Strickland (1878) or ‘*The Merton Rules*’ by Allen (1897). However, more decades were needed to establish a consistent and official set of principles. Those rules are now widely accepted by the international scientific community and described in the International Code of Zoological Nomenclature (ICZN 1961). The idea of the Code was best expressed by J. C. Bradley in the preface to the first ICZN edition:

*Like all language, zoological nomenclature reflects the history of those who have produced it, and is the result of varying and conflicting practices. Some of our nomenclatural usage has been the result of ignorance, of vanity, obstinate insistence on following individual predilections, much, like that of language in general, of national customs, prides, and prejudices. Ordinary languages grow spontaneously in innumerable directions; but biological nomenclature has to be an exact tool that will convey a precise meaning for persons in all generations*.

The regulations of the Code (based on ICZN 1999) concern among others the system of zoological nomenclature, including the alphabet used in naming taxa and derivation of names. According to the ICZN the name should be compact, euphonious, and memorable. This recommendation was given to avoid situations like those of Dybowski (1926), who proposed a series of names for new amphipod species that were over 30 letters long, with the record-holder having 52 letters – *Gammaracanthuskytodermogammarus loricatobaicalensis*; these names were later invalidated. Except for these technical and linguistic recommendations, it seems that the Code gives the taxonomist *carte blanche* in creating new names. The main limitation for authors is that the process of naming new taxa should not cause offence. This clause is fully justified as the history of zoological taxonomy includes cases when etymology was used for inappropriate purposes, e.g., the case of Wilhelm Blandowski, whose descriptions of new species of fish were insulting members of the Philosophical Institute of Victoria who were in conflict with Blandowski (Kean 2005).

Free rein in choosing a name is of significance when describing a large series of taxa. There have been cases when an author simply used ordinal numbers as specific names of new species (Spencer 1969). Many other inspirations are hidden behind the taxonomic names, starting from the most obvious such as general morphology or type locality, through various word-plays (e.g., anagrams or acronyms) and names dedicated to politicians, musicians or comic book characters. Here we make the review of the variety of inspirations for zoological names and discuss some of the most important queries associated with the process of naming and recognition of names.

## Form and function

In the history of taxonomy, the most common animal names are probably those reflecting species morphology, habitat and sometimes even behaviour. Names referring to morphological features can be further divided into several subcategories such as size – one of the largest beetles ever described is *Titanus giganteus* (Linnaeus 1771). A name may also reflect the colour of the animal (the black garden ant *Lasius niger*) or a specific pattern present on the body of the described taxon, like zigzags, dots or stripes (the cowry *Cypraea ziczac* (Linnaeus 1758)).

An inspiration for the name can be a shape of a whole animal e.g., hammer-like bivalves from the genus *Malleus* (Lat. Hammer) (Lamarck 1799); or its appendages, e.g., the tanaidacean *Apseudes batillus* that was named after its wide, shovel-like rostrum (Lat. *batillus* – shovel) (Bamber 2007).

Some of the names describe a species habitat as for the synanthropic spider *Tegenaria domestica* (Lat. *domesticus* – belonging to the house) (Clerck 1757) or in the case of parasites the name of the host e.g., louse *Pediculus humanus* (Linnaeus 1758). Names may reflect life strategies and behaviour, for example the shell-inhabiting tanaidacean *Pagurapseudes inquilinus* from the Latin *inquilinus*, meaning a tenant or lodger (Bamber 2007) and *Macrolabrum mansoris* – from *mansoris* – guest or sojourner (Bamber 2009).

In many cases names provide an appropriate impression of a particular species but there are examples when the name can be misleading or reveals some unusual circumstances that led to the name. One of those unusual cases is that of the New Guinean Greater Bird–of–paradise. Its scientific name given by Linnaeus is *Paradisea apoda* (Linnaeus 1758) and direct translation of its specific Latin epithet means ‘legless’. The specimens available for description indeed did not have legs as members of the tribes who collected the specimens had removed the legs during preparation. Lack of this essential information led to further theories that this bird was a visitor from paradise, spending its entire life in the air.

As it was mentioned above many names can literally describe some characters of the species. Many others are a type of metaphor that shows the intuitive leap between the species and the name. Some of those names are clearly anthropomorphic. This attribution of human form fits into our need to generate assumptions that animals share some of their physical or mental capacities with humans. Inspirations described in this category are often the taxonomist’s personal response to the animal. For example the name of the beetle with an unusual head shape *Agathidium akallebregma* (Miller and Wheeler 2005) can be translated as ‘ugly face’, while the name of the tanaidacean *Pseudoleptochelia anorexia* is associated with its slender morphology (Bird and Bamber 2000). The physical characters might also generate some sort of assumption about mental features. For example the tanaidacean species *Pseudoleptochelia ebriosus* characterized by a presence of red eyes has a name derived from the latin word for drunkard (Bamber and Bird 1997).

## You name the place and I will be there

Knowledge about the distribution of species is very important for the understanding of their biology and ecology. No wonder that there is a series of species named after their type locality or range. The name may point directly the place where the animal was found e.g., the amphipod *Gammarus varsoviensis* collected in the oxbow lake near Polish capital city – Warsaw (Jażdżewski 1975) or a wider geographic area e.g., *Colossendeis fijigrypos* – pycnogonid from Fiji (Bamber 2004). Sometimes the inspiration may also reveal the sense of humor e.g., the name of pycnogonid Pallenopsis (Pallenopsis) desperado (Bamber 2005) is a pun on the French ‘d’Esperance’ (of Esperance – the type locality).

## Gods and demons

Since the beginning of the binominal system, zoological nomenclature has been influenced by mythologies and religions. Inspirations from Greek mythology were and probably still are in the lead mostly because of numerous names (mostly butterflies) created by Linnaeus and other pioneers of taxonomy (Tolman and Levington 2004). This situation was most probably associated with the classical education in those days. Linnaeus studied Greek and literature, and mythology of the ancient Greece was the basis of education in many European countries (Huxley 2007). One of the innumerable examples from this category is that of the Little Owl that owes its Latin name – *Athene noctua*, to the Greek goddess Athena (Scopoli 1769). In this case it is easy to track the association between the species and its mythic archetype as both Athena and owls are symbols of wisdom; moreover the Little Owl was sacred to Athena. A further example is the name of a brittlestar, *Gorgonocephalus caputmedusae*. The name can be translated as ‘head of Gorgon’ and ‘head of Medusa’ (Medusa was one of the gorgons with serpent–like hair) and reflects multifurcate and curled arms present in this species (Linnaeus 1758, Leach 1815). Furthermore, the name of the small nepticulid moth Pectinivalva (Casanovula) minotaurus, a species characterized by expanded antennae, refers to the Minotaur’s horns (Hoare and Nieukerken 2013).

Hellenic culture of the ancient Greece has also a great influence on forming of the Judaeo-Christian tradition, which is also present in zoological nomenclature. The Mediterranean bivalve *Arca noae* (Linnaeus 1758), owes its name to its similarity to the Biblical Ark. The spider name *Aptostichus asmodaeus* was dedicated to Asmodeus (King of Demons), a fallen angel mentioned in the Book of Tobias, in reference to the type locality, Mount Diablo State Park (Bond 2012). There are also some taxonomic names derived from various versions of the Devils name, starting from the original Hebrew term Satan, through Lucifer, which refers to planet Venus and ending with Mephistopheles, from a German Faust legend. A blind catfish associated with aquifers was named *Satan* (Hubbs and Bailey 1947), while a nematode that was described as the deepest ever recorded land animal (found in terrestrial deep subsurface of South Africa, 2.2 miles underground) was named *Halicephalobus mephisto* (Borgonie et al. 2011). The name of Lucifer (Lat. *lux* – light and *ferre* – to carry) is often used as a name for animals that have abilities to produce light e.g., the dragonfish *Lucifer* (Döderlein 1882) but also in more direct sense, like in case of tanaidacean *Pakistanapseudes lucifer* (Błażewicz-Paszkowycz and Bamber 2012) which was named after the Devil, owing to its bifurcated claws on pereopods.

Other beliefs are represented in names mainly from Scandinavian mythology, e.g., some common Eurasian butterflies are named after Thor and Frigga – Thor’s fritillary (*Boloria thore*) and Frigga’s fritillary (*Boloria frigga*) respectively (Hübner 1803). One of the most recognizable examples of names inspired by religion from the Indian subcontinent is *Stegodon ganesa*, a Pliocene mammal closely related to extant elephants. The species was found in India and its specific name regards to mythical Ganesa, one of the most important gods from Hindu pantheon, usually shown with the head of elephant (Saegusa 1987).

## Monsters and cryptids

Legendary or mythical animals can be found in civilizations from various regions of the world and taxonomists find inspirations from them and the related field of cryptozoology. One of the best known is the myth of the Yeti – a mysterious anthropoid cryptid inhabiting the Himalayan Mountains; a carabid beetle was named *Agra yeti* (Erwin 1982) because of its big feet.

Monsters that suck blood and the vital power from people are known in many legends and myths. Some of these demons sucked vital powers from men, and one of the oldest, Empusa from Ancient Greece, lent its name to a praying mantis *Empusa* (Illiger 1798). This myth ‘evolved’ to more modern versions, including the current cultural obsession with zombies and vampires. Three genera of South-American bats were named *Vampyrodes*, *Vampyrops*, *Vampyressa* (Thomas 1889, 1900). A deep-sea cephalopod, *Vampyroteuthis infernalis*, is even more terrifying, because its name means ‘vampire squid from hell’ (Chun 1910).

## The first muse

Aoede was the first original Boeotian muse, a muse of songs and voice, and we can say that music has inspired humans since the ancient times. Scientists have often declared their enthusiasm for music, both as listeners and performers. Albert Einstein said that “*life without playing music is inconceivable*” and he often declared that music was an undeniable help in his work. He is not alone, with a paper concerning amphipod reproductive traits (Grabowski et al. 2014) being dedicated to the Norwegian duet Ylvis. The authors wrote in the acknowledgements that Ylvis “*songs and videos kept us in a continuous good mood when analysing the data and writing the manuscript*”. It is unsurprising that many taxa have been named after famous musicians, bands or even music styles and genres, but it is remarkable that these names represent so diverse range of inspirations, from well known pop classics (The Beatles – polychaete worm *Bushiella beatlesi*, Michael Jackson – fossil paguroid *Mesoparapylocheles michaeljacksoni*) (Rzhavsky 1993, Fraaije et al. 2012) through major progressive rock bands (Pink Floyd – spider genus *Pinkfloydia*) (Dimitrov and Hormiga 2011), jazz icons (Miles Davis – trilobite *Milesdavis*) (Liberman 1994) and local folk ensembles (Argentinean band Los Chalchaleros – caviomorph rodent *Salinoctomys loschalchalerosorum*) (Mares et al. 2000). Not excluded are classical music (Ludwig van Beethoven – isopod crustacean *Gnathia beethoveni*) (Paul and Menzies 1971) and obscure avant-garde genres known to only small groups of enthusiasts (e.g., Dutch post-punk group The Ex – gastropod *Depressizona exorum*) (Geiger 2003).

Most of these names explicitly refer to the person or group; however some of the linkages between music and the morphology or biology of a new taxon result in intriguing names. The generic name of the ichneumonid wasp *Metallichneumon neurospastarchus* refers to the heavy metal band Metallica. This wasp parasitizes sphingid moth caterpillars and *neurospastarchus* derives from the Greek words *neurospasta* (puppet) and *archos* (ruler), linking the famous Metallica album ‘Master of Puppets’ with “*mindless nature of lepidopterous larvae*” (Sime and Wahl 2002).

## Reading and writing

Writing is an indispensable element of scientific work. If done well, it provides the most effective method of communication with other scientists. Yet, according to a well-known reporter Ryszard Kapuściński (2008) to write one single page one probably needs to read at least 100 pages first. In consequence, all one’s previous reading (including favourite popular fiction) has a great influence on writing style, both in life and in scientific texts. Taxonomic inspirations associated with book titles, famous authors and fictional characters from literature are numerous amongst taxonomic eponyms. It is worth mentioning that some writers were at the same time professional scientists. Vladimir Nabokov is famous for his novel "Lolita", however the name of the lycaenid butterfly *Nabokovia* (Hemming 1960) was dedicated to him not because of his writing, but due to his passion for taxonomic studies on butterflies. Nabokov was a curator of the lepidopteran collection at Harvard University’s Museum of Comparative Zoology in the 1940s and he even published articles concerning this subject (e.g., Nabokov 1944). Many names of butterflies are derived from the main protagonists in his novels, such as *Madeleinea lolita* (Balint 1993).

In the early twentieth century the American entomologist Alexandre Arsène Girault dedicated some of the names of the wasps he studied to influential writers such as William Shakespeare (*Shakespearia*) (Girault 1928), Johann Wolfgang von Goethe (*Goetheana*) (Girault 1920) and Henry Wadsworth Longfellow (*Tineobius longfellowi*) (Girault 1923). In the years since then, the names of writers and books used in taxonomy have changed notably.

Many names have been created in honour of the British author of fantasy novels, Terry Pratchett, especially after the characters from his Discworld series. Roger Bamber was especially fond of these books and named 30 species of ‘Discworld’ tanaidaceans, three of which are *Apseudes atuini* – derived from Great A’Tuin, the giant space turtle on whose back four elephants hold up the Discworld, *Tanaella dongo* – Crocodile Dongo, a creature that runs a pub in the town of Dijabringabeeralong, on the Last Continent, and *Bathytanais greebo* – Nanny Ogg’s cat Greebo (Bamber 2005). There are also many species named after characters created by J. R. Tolkien in his trilogy *Lord of The Rings*, including the tanaidacean, *Gollumudes*, from Gollum who dwelt in damp muddy caves (Bamber 2000), and a beetle, *Pericompsus bilbo*, whose hairy feet refer to the hobbit Bilbo Baggins (Erwin 1974). New bestselling classics, like J. K. Rowling’s *Harry Potter* series of novels, inspired the names such as the dinosaur *Dracorex hogwartsia* (Bakker et al. 2006). We can expect that, with the appearance of new ‘bestsellers’, the number of new taxa with names derived from literature will continue to increase.

## The big screen

Actors, directors and movie characters have been an inspiration for some taxonomists. Former governor of California and actor, Arnold Schwarzenegger, was commemorated in the name *Agra schwarzeneggeri* since males of this species have thickened thighs, which are similar to his well-muscled limbs (Erwin 2002). The name *Agra liv* was dedicated to the Hollywood actresses Liv Tyler. As described by the author, she survived the impending apocalypse in the Armageddon movie, but devastation of tropical rainforests may also lead to an ‘Armageddon’ for this small beetle (Erwin 2002). People from other side of the camera also have been commemorated in taxonomy. Director Steven Spielberg was honoured for his three Jurassic Park movies with a pterosaur species named *Coloborhynchus spielbergi* (Veldmeijer 2003).

Movie characters, rather than the actors themselves, sooner or later feature in zoological names. Terminator was a deadly robot from the future and authors who described the African spider *Hortipes terminator* found similarities in the appearance of its pedipalps to Terminator’s weapon (Bosselaers and Jocqué 2000). Batman, masked defender of Gotham City, is a pop culture icon. The name of the North-American fish *Otocinclus batmani* (Lehmann 2006) is associated with a black spot on its caudal fin, the shape being similar to the image generated by the searchlight used by Commissar Gordon to summon Batman. Perhaps inevitably, the popular movie series *Star Wars* by George Lucas was also an inspiration for many taxonomists. Master Yoda’s ears were so similar to appendages on the sides of the head of the small parasitic isopod *Albunione yoda*, that authors gave its name after this Jedi (Markham and Boyko 2003).

Some characters from cartoons have also inspired taxonomists. According to the authors, the male aedeagus of a beetle *Adelopsis dumbo* look like the large and floppy ears of the elephant Dumbo (Gnaspini and Peck 2001).

## From Aristotle to Darwin

Many species and genera have been named in honour of scientists. These have appeared since the time of the ‘Father of modern taxonomy’ Carolus Linnaeus. He named the Common Shag *Phalacrocorax aristotelis* (Linnaeus 1958) to honour Aristotle. The list of scientists appreciated by their colleagues and followers is very long, and some of them were honoured many times. We can even say that the number of taxa named after a researcher can be considered a signifier of their importance in the history of science. Pioneers of taxonomy Linnaeus and Lamarck were commemorated in many taxonomic names, e.g., the snake *Calamaria linnaei* and polychaete worm *Pomatoceros lamarckii* (Boie 1827, Quatrefages 1866).

Nevertheless, judging from the zoological nomenclature Charles Darwin has been probably the most inspiring and influential scientist. There are over 300 taxa from nine phyla named after him (Miličić et al. 2011). These include over 250 species, many genera (e.g., ostracod *Darwinula* or hemipteran *Darwinysius*) but also some higher ranks like the ostracod Infraorder Darwinulocopina (Sohn 1988). His work “On the Origin of Species” became the fundamental text for biologists but it was also highly contested and criticized, mostly because of the lack of the true understanding of his ideas. So, as we might expect, a person who has contributed much to improve the understanding of evolutionary biology would also be memorialized with a taxonomic name. A cyprinid fish (*Dawkinsia*) was named after Richard Dawkins, author of the popular science books "The Selfish Gene" and "The Blind Watchmaker", for “*his contribution to the public understanding of science and, in particular evolutionary science*” (Pethiyagoda et al. 2012).

## Politicians and the powerful

Politicians always have great influence on peoples’ lives for good or bad. A large group of scientific names commemorates former presidents of the United States, including Abraham Lincoln (the parasitic wasp *Lincolna* (Girault 1940)) and Franklin Delano Roosevelt (the amphipod *Neomegamphopus roosevelti* (Shoemaker 1942)). From more recent times, George W. Bush, Vice President Dick Cheney, and Secretary of Defense Donald Rumsfeld were honoured in the names of Leiodidae beetles: *Agathidium bushi*, *Agathidium cheneyi*, and *Agathidium rumsfeldi* (Miller and Wheeler 2005).

The emperors of Japan were treated with great dignity and respect, and were even considered deities. Hirohito, the longest-reigning emperor (1926–1989) was also a marine biologist and inspired the name of a sea gastropod *Rotaovula hirohitoi* (Cate 1973). A beetle, *Aegomorphus wojtylai*, was recently dedicated to a former head of the Catholic Church, Karol Wojtyła (Pope John Paul II) (Hilszczański and Bystrowski 2005).

There is clearly a negative aspect to “political taxonomy”. The German entomologist Oscar Scheibel (1937) honoured the leader of the National Socialist German Workers’ Party, Adolf Hitler, by describing *Anophthalmus hitleri* – a species of carabid beetle from the Balkans. This endemic insect occurs only in five caves in Slovenia, and is now a victim of Hitler’s notoriety as specimens are being illegally collected as ‘memorabilia’ of the Third Reich. The restricted range and very specific habitat of this endemic beetle has pushed it to the brink of extinction.

## Brands and sponsors

Some of the most widely known international brands, or companies, are reflected in taxonomic names. Probably the world’s best known drink manufacturer, Coca–Cola©, was included in the name of the wasp *Oxybelus cocacolae* (Verhoeff 1968), although more subtle links between companies’ and species’ names have been made. A species of ant from Madagascar, *Proceratium google*, named in recognition of Google©, has a great “*ability to hunt down obscure prey* [Sic!]” (Fischer 2005).

Financial and logistic support to scientific expeditions are also rewarded in the names given to new species: a team of palaeontologists found and described a new Palaeocene mammal, *Roberthoffstetteria nationalgeographica*, (Goin et al. 2003) during an expedition supported by the publisher of the National Geographic magazine. Conservation efforts can be an outcome of cooperation between business and nature-protection agencies: a model example happened in Bolivia where the online casino GoldenPalace.com paid hundreds of thousands of dollars for the rights to name the new species of primate, *Callicebus aureipalatii* (Lat. *aureus* – golden and *palatium* – palace), in the Madidi National Park. Moreover, the company decided to pay an annual subsidy for this national park (Wallace et al. 2006).

## Playing with words

In the introduction we said that taxonomists have (almost) free rein in choosing the name for new taxa and this no-restriction policy is most evident in this category. It includes various types of word plays, figures of speech and rhetorical devices. In most cases it is hard to say that there is any significant ‘inspiration’ hidden behind these names. Some of the examples from this category might be called controversial and may even bend the rules of the ICZN.

Tautonyms are names where the specific epithet is repeated after the genus. They were widely used for, and often characterize, common European animals such as the Roe Deer – *Capreolus capreolus*, or Fox – *Vulpes vulpes* (Linnaeus 1758). This tautonymy has a certain euphonious effect, in contrast to some true ‘tongue twisters’ such as the nematode *Xyzzors* (Inglis 1966) or snail, *Zyzzyxdonta* (Solem 1976).

An early example of wordplay is the taxonomic work of English marine biologist Elford Leach. In his 1818 monograph he described a series of isopod genera that were anagrams of ‘Caroline’ or ‘Carolina’: *Anilocra*, *Cirolana*, *Conilera*, *Lironeca*, *Nerocila*, *Olencira*, and *Rocinela* (Leach 1818). Later this idea was picked up by Hansen (1890) who described *Alcirona* and *Lanocira*, and Nierstrasz (1931) who described *Orcilana*.

Palindromes, i.e. words that can be read the same forward and backward, are not so common in zoological nomenclature and are usually applied to genus names e.g., the bethylid hymenopteran *Afgoiogfa* (Argaman 1988), and more rarely in species names, as for the syrphid fly *Xela alex* (Thompson 1999).

Acronyms are often used to honour an institution or project that was involved in collecting the material or financially supported the taxonomic studies e.g., *Pseudotanais soja* (Błażewicz et al. 2013) a tanaidacean species collected during expedition of SoJaBio (Sea of Japan Biodiversity Studies). Błażewicz-Paszkowycz and Bamber (2013) named tanaidacean *Acinoproskelos vermes* after Latin plural for “worms”, being both a reference to the parasitic nematodes within the type specimen, and also the acronym of the World Register for Marine Species (WoRMS). There is, however, an example of an acronym–name that probably appeals to those taxonomists spending a lot of time in the laboratory – *Afropolonia tgifi* (Thank God it’s Friday) (Goff 1983).

Finding good names when a large series of taxa has to be described can be time consuming and various ‘tricks’ are sometimes used to facilitate this. Taxonomists might use the same word stem and supplement it with a different prefix or suffix. This was used by the British, American entomologist Kirkaldy (1904), who proposed numerous hemipteran names with the Greek stem ‘-chisme’ including: *Marichisme*, *Peggichisme*, and *Ochisme*. At first glance this series is unremarkable but phonetically in English it results in the creation of short phrases: ‘Mary kiss me!’, ‘Peggy kiss me’ and ‘O kiss me!’. By a decision of the Zoological Society of London, Kirkaldy was criticized in 1912 for this frivolity but it seems that it has not discouraged other taxonomists from similar usage, as Evenhuis (2002) described a fossil fly in the genus *Carmenelectra* (named after the singer, actress and model) with the specific name ‘*shechisme*’.

Another method for naming dozens of species was used by Kearfott (1907) who described a series of new moths from the genus *Eucosma* with names created in the same way distinguished only in their consonants: e.g., *Eucosma bobana*, *Eucosma cocana*, *Eucosma dodana* or *Eucosma fofana*. Spencer (1969) described several agromyzid flies from the genus *Ophiomyia* using ordinal numbers for the specific names: *Ophiomyia prima*, *Ophiomyia secunda*, *Ophiomyia tertia* and so on. Riedel et al. (2013) also did not have easy task as he needed to find names for 101 species of curculionid beetles. In this case, the Papua New Guinea Telephone Directory, appeared to be helpful and Riedel use the names of people found there as the stem with the Latin suffix ‘-*orum*’ e.g., *Trigonopterus hitoloorum*, *Trigonopterus kanawiorum*, *Trigonopterus koveorum* or *Trigonopterus lekiorum*.

There are many names that are created from Latin words that are sometimes meaningful taking into account the taxonomic context e.g., *Sphenoptera incerta* (unsure) (Jakovlev 1887) or *Leptura dubia* (Scopoli 1763) (doubtful). Names may also present an unusual combination of words as in the African cicada, *Imbecilla cretinica* (Dworakowska 1974).

## Concluding remarks

While names are often derived from the attributes of a species (e.g., its morphology or behaviour) it may also be a result of subjective state of taxonomist’s mind at the time of naming, including those who were assisted by the music of a certain band or composer. Sometimes a curious name is used simply to get other people interested in a given taxon, but more often than not it has some stronger basis. Nevertheless, it is generally very difficult to analyse all types of taxonomic inspiration since we have to follow only the information provided in the etymology section of cited papers, and we do not wish speculate about the taxonomist inspirations even if the name generates some questions about the author’s decision.

Continuous findings of new taxa results in a obvious necessity of naming them. The name is very important because it allows to communicate about a given species with other scientists. Therefore, it seems that it is also important to ask what we can learn from a name? This function of the name may show a need for accuracy in scientific naming. Some people could say that the name itself should be as informative as possible, and give us also some basic information about the species. Taxonomist may use a very simple words to describe the taxon or try to be more subtle in their inspirations like for example link the species morphology with description of a mythological creature or god (Winston 1999).

On the other hand naming process reflects also fads and trends present in the modern world. Those fads are present in all languages and it is not surprising that are also visible in taxonomy. There are numerous names that are very attractive but cannot give us any meaningful message, that is significant from the biological point of view. At the same time the name may educate us about culture, history or politics, however it is important to ask if this is really the function of taxonomic eponym? It is probably not, nevertheless, it is worth mentioning that naming of large series of morphologically similar new species that we know almost nothing about often do not allow to maintain the informative function of the names.

Naming process may also result in problems of ethical nature. For example the name of the sponsor, company or a certain politician commemorated in a taxonomic name may caused (often not intended) questions associated with the policy of those companies or accusations of showing allegiance. However, one could say also that if the sponsoring allows for a development of knowledge it is still consistent with the ethical standards. In some cases the name can be also a political statement because according to the etymology sections taxonomist approve the politics of presidents and ministers which are mentioned in the taxonomic names. On the other hand it is worth to remember that it may result in completely opposite reactions of other researchers. Moreover, we cannot be sure that we will approve also all future decisions of the active politician. Finally we may also consider the fact that taxonomic names may also completely lose their meaning, like in case of *Aegomorphus wojtylai* which was synonymised just three years after description (Hilszczański 2008).

Analysis of species names raises questions about our understanding of names. Some names may lose impact or ‘recognition’ over the years. We do not know if we will be able to comprehend the inspiration associated with the Star Wars saga 200 years from now, and knowledge of music bands like The Beatles or authors like Longfellow might not be present among many biology students today. Nevertheless, taxonomic names persist and will still be used by other taxonomists even if their etymology will not be clear for everyone or becomes outdated. Taking these cultural changes into account it is interesting to ask if it is appropriate that scientists should have such a wide choice of names. We did not intend to find the final answer to this question here, but propose that each taxonomist should consider it during their own work. We can only repeat after Brown (1956) that invention of names is an art and simplicity is often the most appropriate choice.
